# Editorial: Understanding Developmental Dyslexia: Linking Perceptual and Cognitive Deficits to Reading Processes

**DOI:** 10.3389/fnhum.2016.00140

**Published:** 2016-03-31

**Authors:** Pierluigi Zoccolotti, Peter F. de Jong, Donatella Spinelli

**Affiliations:** ^1^Department of Psychology, Sapienza University of RomeRome, Italy; ^2^Neuropsychology Unit, IRCCS (National Institute for Research and Treatment) Fondazione Santa LuciaRome, Italy; ^3^Department of Child Development and Education, University of AmsterdamAmsterdam, Netherlands; ^4^Department of Human Movement Sciences and Health, University of Rome “Foro Italico”Rome, Italy

**Keywords:** dyslexia, reading

The problem of causation has proven particularly elusive in the case of developmental dyslexia (DD). The field has been dominated by very general hypotheses, such as the idea that DD is caused by a phonological deficit and/or an impairment of the magnocellular pathway. Results are contrasting and causal unidirectional links have not been persuasively demonstrated.

Some studies in the Research Topic (RT) re-examine these general hypotheses from the critical perspective of more selective predictions. Others focus on less general deficit hypotheses and stay closer to reading by investigating specific aspects of the reading process such as orthographic learning ability or the ability to deal with multiple-stimulus displays. Studies benefit from new research paradigms as well as new information from research areas such as neuroimaging or genetics. Below, we sketch the general questions tackled by these studies.

## Orthographic learning

Unlike standard studies, which provide a static snapshot of reading performance, learning studies allow asking questions about how children acquire words. Kwok and Ellis and Suárez-Coalla et al. capitalize on the observation that presenting pseudo-words in repeated blocks reduces the size of the length effect (Martens and de Jong, 2008). Results are generally in keeping with the idea that dyslexic children are impaired in forming orthographic representations and continue to use sublexical reading during the course of learning. Wang et al. examine orthographic learning as a function of specific (phonological and surface) individual reading profiles using a new learning task. They point out that orthographic knowledge predicts orthographic learning over and above phonological decoding and that orthographic impairment is actually more important than phonological impairment in the learning of new words.

## Dealing with multiple stimuli

Current models of reading focus on single word reading but are commonly extended to explain reading in more natural contexts i.e., text reading. One potentially important way to understand DD is to contrast reading of single vs. multiple stimulus displays.

Control children read multiple items faster than single items; this indicates that they process the next visual stimulus while uttering the current target; dyslexic children fail to show such an advantage (Zoccolotti et al., 2013). A paradigm that captures the need to smoothly integrate all the various sub-components involved in reading (except for orthographic analysis) is rapid automatized naming or RAN (Denckla and Rudel, 1976). Two studies capitalize on this observation (Gasperini et al.; Zoccolotti et al.) and point out the importance of considering the multicomponential nature of reading to obtain a full description of DD.

## From attentional hypotheses of dyslexia to the hypothesis of pre-lexical loci of the disturbance

Various attentional deficits have been identified in dyslexic children (e.g., Vidyasagar and Pammer, 2010), but their precise role is still underspecified.

Kezilas et al. report the possible causes of letter position dyslexia. Their evidence is in keeping with the idea of a deficit in the coding of letter positions at the orthographic-visual analysis stage of reading. Lukov et al. note various forms of double dissociations between reading and attention deficits: attention categories, such as sustained, selective, orienting and executive attention functioning, do not effectively map into reading difficulties. Lobier et al.'s study stems from the visual attention (VA) span deficit hypothesis of DD (Bosse et al., 2007); it shows dysfunctions in a categorization task for multiple (but not single) alphanumeric (and non-alphanumeric) stimuli.

Overall, attentional deficits are clearly dissociated from reading deficits; thus, specific hypotheses (such as the VA span deficit hypothesis) are needed to explain reading related attentional deficits.

## Meta-analyses of neuroimaging studies

There has been a dramatic increase in studies on reading that are based on imaging paradigms. Paulesu et al. report a meta-analysis of 53 neuroimaging studies of DD. When activations are analyzed, those of dyslexic subjects (but not controls) indicate a distributed set of local malfunctions in “associative” regions normally involved in more than one behavior/cognitive domain. Richlan's meta-analysis focuses on whether different manifestations of dyslexia across languages are associated with different functional neuroanatomical manifestations. The effect of orthography is a relevant general question, which is underscored also in other papers (Angelelli et al.; Lukov et al.; Kezilas et al.). In particular, Angelelli et al. demonstrate that even in a very regular language (such as Italian) morphological information is a useful resource for both reading and spelling. Future neuroimaging studies should be usefully informed by the articulated conclusions of these meta-analyses.

## Biological indicators of learning and learning deficits

Schiavone et al. show that two EEG biomarkers recorded in 3-year-old children from families at risk of dyslexia correlate with performance in various tasks including reading fluency, phonological awareness, orthographic knowledge and RAN assessed at 9 years of age. Hasko et al. investigate whether the EEG neurophysiological profile of children with dyslexia before intervention predicts the success or failure of future training. Longitudinal and intervention studies in dyslexia that include biomarkers are rare and important: these two studies indicate the growing interest in the biological indicators of dyslexia and learning deficits.

## The magnocellular hypothesis of dyslexia

A well-known hypothesis sees DD as due to a magnocellular deficit (Stein, 2001). Possibly indicating little interest in this theoretical framework, no work in the RT directly tests this hypothesis. However, in their extensive meta-analysis Paulesu et al. note the absence of any deficit in the V5/MT area (the core magnocellular region) in dyslexics. A key area of investigation in the magnocellular hypothesis is the study of eye movements (Boden and Giaschi, 2007). The study by Gagl et al. confirms that slow readers process words by means of serial decoding but have corrective processes similar to those of proficient readers after landing at unfavorable positions within a word. Overall, these findings are not in keeping with the notion that magnocellular dysfunction generates DD.

## The phonological hypothesis of dyslexia: from general to specific tests of the hypothesis

Much research on DD is based on “*the pivotal role of phonemic awareness as a predictor of individual differences in reading development*” (Melby-Lervag et al., 2012). However, correlation between abilities does not mean that a deficit in phonological abilities *causes* a reading deficit as it is difficult to exclude the alternative possibility, i.e., that the lack of reading experience associated with DD causes poor performance on meta-phonological tests. Some studies examine the relationship between phonology and dyslexia through more tuned questions with respect to questions than those adopted in previous research.

Leong and Goswami move within the oscillatory temporal sampling framework of dyslexia (the reader can also find relevant information on a recent RT; Goswami et al.). Law et al. examine whether auditory, speech perception, and phonological skills are tightly interrelated or contribute independently to reading. Gimenez et al. evaluate the correlation between reading and handwriting at the beginning of formal handwriting instruction with the hypothesis that handwriting and reading may initially share a common neural mechanism.

A variant of the phonological hypothesis is that DD is due to an inability to bind orthographic and phonological information (Blomert, 2011). Marinelli et al. test this hypothesis by contrasting it with the idea that the reading deficit may be due to a deficit at the pre-lexical graphemic level.

Within this general framework, a recent hypothesis refers to a deficit in learning serial order information either in the consolidation phase of learning (Szmalec et al., 2011) or at the STM level (Martinez Perez et al., 2012, 2013). Staels and van den Broeck consider this latter possibility and provide evidence against this view.

The idea that dyslexia can be ascribed to many factors raises the question of co-morbidities in the genesis of the behavioral disturbances shown by children with dyslexia. Consistently with previous data (e.g., Brizzolara et al., 2006; Chilosi et al., 2009), Lorusso et al. demonstrate the important modulating role of a previous language delay on DD.

## Modeling dyslexia within the comorbidity perspective

Several of the studies in the RT point out the multi-factorial nature of reading deficits. A theoretical perspective which is particularly suited to this aim is that reading (and more generally learning) disorders can be effectively described within a comorbidity perspective (Pennington, 2006). Drawing on Pennington's model, as well as on Plomin and Kovas's (2005) generalist genes hypothesis of learning (dis)abilities, van Bergen et al. propose the intergenerational multiple deficit model in which both parents confer liability via intertwined genetic and environmental pathways.

## Final remarks

The main tendency of the studies presented in the RT is to move away from broad, general hypotheses of the disorder, such as phonological or attentional ones, and to consider hypotheses that on the one hand are more explicit about the perceptual and linguistic processes specifically involved in reading (such as orthographic learning ability or the ability to deal with multiple-stimulus displays) and on the other try to link these mechanisms to a proximal analysis of the reading processes (as in the analysis of letter position dyslexia). Overall, dyslexia emerges as a multiple-cause deficit and in this light future research should be oriented toward considering the problem of comorbidity.

## Author contributions

All authors gave a similar contribution. PZ: wrote part of the first draft of the paper. DS: made several changes to various versions of the manuscript. PdJ: made several changes to various versions of the manuscript.

### Conflict of interest statement

The authors declare that the research was conducted in the absence of any commercial or financial relationships that could be construed as a potential conflict of interest.
